# Has Cox-2 a prognostic role in non-small-cell lung cancer? A systematic review of the literature with meta-analysis of the survival results

**DOI:** 10.1038/sj.bjc.6603226

**Published:** 2006-06-20

**Authors:** C Mascaux, B Martin, M Paesmans, T Berghmans, M Dusart, A Haller, P Lothaire, A-P Meert, J-J Lafitte, J-P Sculier

**Affiliations:** 1 Department of Intensive Care and Thoracic Oncology, Institut Jules Bordet, Centre des Tumeurs de l'Université Libre de Bruxelles, B-1000 Brussels, Belgium; 2 Data Centre, Institut Jules Bordet, Centre des Tumeurs de l'Université Libre de Bruxelles, B-1000 Brussels, Belgium; 3 Department of Nuclear Medicine, Institut Jules Bordet, Centre des Tumeurs de l'Université Libre de Bruxelles, B-1000 Brussels, Belgium; 4 Department of Pathology, Institut Jules Bordet, Centre des Tumeurs de l'Université Libre de Bruxelles, B-1000 Brussels, Belgium; 5 Department of Surgery, Institut Jules Bordet, Centre des Tumeurs de l'Université Libre de Bruxelles, B-1000 Brussels, Belgium; 6 Chest Department, CHU Calmette, F-59037 Lille, France

**Keywords:** COX-2, lung cancer, meta-analysis, systematic review, survival, prognostic factor

## Abstract

Cyclooxygenase-2 (COX-2) is overexpressed in lung cancer, especially in adenocarcinoma (ADC). Our aim was to determine the prognostic value of COX-2 on survival in patients with lung cancer. Studies evaluating the survival impact of COX-2 in lung cancer, published until December 2005, were selected. Data for estimation of individual hazard ratios (HR) for survival were extracted from the publications and combined in a pooled HR. Among 14 eligible papers, all dealing with non-small-cell lung cancer, 10 provided results for meta-analysis of survival data (evaluable studies). Cyclooxygenase-2 positivity was associated with reduced survival, improved survival or no statistically significant impact in six, one and seven studies, respectively. Combined HR for the 10 evaluable studies (1236 patients) was 1.39 (95% confidence intervals (CI): 0.97–1.99). In stage I lung cancer (six evaluable studies, 554 patients), it was 1.64 (95% CI: 1.21–2.24). No significant impact was shown in ADC. A slight detrimental effect on survival in patients with lung cancer is associated with COX-2 expression, but the statistical significance is not reached. This effect is statistically significant in stage I, suggesting that COX-2 expression could be useful at early stages to distinguish those with a worse prognosis.

Lung cancer is a major cause of death despite diagnostic and therapeutic improvements. The overall 5-year survival rate is around 10% (Boyle and Ferlay, 2005). Some independent prognostic factors for survival have already been identified. They include, for small cell lung cancer (SCLC): disease extent and performance status (PS) (Paesmans *et al*, 2000); for non-small-cell lung cancer (NSCLC): PS, stage and, with lower impact, age, sex and weight loss (Paesmans *et al*, 1995; Strauss, 1997). The biological factors involved in carcinogenesis should also be considered as potential survival prognostic factors. Some of them, like angiogenesis and factors reflecting proliferative state, have already been identified in patients with lung cancer (Kanters *et al*, 1995). In order to clarify the prognostic impact of other biological factors in lung cancer, our group has performed systematic reviews of the literature with meta-analyses. It allowed us to show that VEGF (Delmotte *et al*, 2002), microvessel density (Meert *et al*, 2002b), EGFR (Meert *et al*, 2002a), HER-2/Neu (Meert *et al*, 2003), Ki-67 (Martin *et al*, 2004), K-Ras (Mascaux *et al*, 2005a) and p53 (Steels *et al*, 2001) have a negative impact on survival, whereas Bcl-2 (Martin *et al*, 2003) is associated with a favourable survival effect, at least when studying their impact in univariate analysis.

Recent attention has been drawn to prostaglandins and cyclooxygenases (COX) with the discovery that colonic polyps in patients with familial adenomatous polyposis (FAP) are decreased after the administration of non-steroidal anti-inflammatory drugs (NSAIDs) (Waddell and Loughry, 1983). Cyclooxygenases are key enzymes in the conversion of arachidonic acid to prostaglandin and exist as two isoforms, COX-1 and COX-2 (Smith and Langenbach, 2001). Cyclooxygenase-1 is constitutively expressed in nearly all cell types and plays a central role in many normal physiological processes, such as cytoprotection of gastric mucosa. *COX*-2 is a highly inducible gene, activated by cytokines, growth factors, phorbol esters, oncogenes and chemical carcinogens (Smith and Langenbach, 2001). Overexpression of COX-2 has been reported in many human malignancies including head and neck carcinomas (Gallo *et al*, 2002; Lin *et al*, 2002), oesophagus (Lagorce *et al*, 2003), colon (Sinicrope and Gill, 2004), breast (Ranger *et al*, 2004), pancreas (Kokawa *et al*, 2001) and prostatic cancer (Edwards *et al*, 2004).

In NSCLC, an increase in COX-2 expression was detected both in adenocarcinomas (ADC) and in squamous cell carcinomas (SQCC), but at a higher level in ADC than in SQCC (Hida *et al*, 1998; Wolff *et al*, 1998; Ochiai *et al*, 1999). Cyclooxygenase-2 expression was also increased in atypical adenomatous hyperplasia, a possible precursor of ADC (Hida *et al*, 1998; Wolff *et al*, 1998; Hosomi *et al*, 2000; Hasturk *et al*, 2002) and in severe dysplasia and *in situ* carcinoma, precursors of SQCC (Mascaux *et al*, 2005b). However, the literature remains controversial about the prognostic value of COX-2 for survival in patients with lung cancer. In order to clarify this question, we performed a systematic review of the literature with methodological assessment and meta-analysis.

## MATERIALS AND METHODS

### Selection of the publications

To be eligible for the systematic review, a study had to fulfil the following criteria: to deal only with lung cancer (any stage or histology), to analyse the association between COX-2 and survival, to assess COX-2 on the primary tumour (not on metastatic tissue or tissue adjacent to the tumour), to have been published as a full paper in English or French. Abstracts were excluded because they do not provide sufficient data to evaluate the methodology of the trial and/or to perform meta-analysis.

Studies were identified by an electronic search on Medline databank and using the following keywords: ‘lung cancer’, ‘lung carcinoma’, ‘lung neoplasms’, ‘lung tumor’, ‘lung tumors’, ‘lung tumour’, ‘lung tumours’, ‘lung adenocarcinoma’, ‘lung squamous’, ‘NSCLC’, ‘non-small cell lung cancer’, ‘non small cell lung cancer’, ‘non-small cell lung carcinoma’, ‘non small cell lung carcinoma’, ‘SCLC’, ‘small cell lung cancer’, ‘small cell lung carcinoma’, ‘cyclooxygenase’, ‘cyclooxygenase-2’, ‘COX-2’. The bibliographies reported in all the identified studies were used to complete this search, which ended on December 2005.

### Methodological assessment

To assess the methodology, each study report was read independently by 10 investigators. The participation of many readers was a guarantee for the correct interpretation of the articles. The methodological evaluation was scored according to the European Lung Cancer Working Party (ELCWP) scale previously published (Steels *et al*, 2001) and applied in other meta-analyses (Delmotte *et al*, 2002; Meert *et al*, 2002a, 2002b, 2003; Martin *et al4*, 2003, 2004; Mascaux *et al*, 2005a). Each item was assessed using an ordinal scale (possible values: 2, 1, 0). A consensus was reached in regular meetings where at least two-thirds of the investigators needed to be present. As the assessed items were objective ones, a consensus was always obtained.

The overall score evaluated several dimensions of the methodology, grouped in four main categories: the scientific design, the description of laboratory methods used to identify COX-2 expression, the generalisability of the results and the analysis of the study data. Each category had a maximum score of 100 points, with a maximal theoretical score of 400 points. When an item was not applicable to a study, its value was not taken into account in the total of the concerned category. The final scores were expressed as percentages, ranging from 0 to 100%, higher values reflecting better methodological quality. Studies included in the systematic review were called ‘eligible’, those providing sufficient data for the meta-analysis ‘evaluable’. To be eligible, studies had to provide univariate survival analysis according to COX-2.

### Statistical methods

A study was considered significant if the *P*-value for the statistical test, comparing survival distributions between the groups with and without COX-2 increase, was <0.05. A study was called respectively, ‘positive’ or ‘negative’ when COX-2 increase was identified as a significant favourable or unfavourable prognostic factor for survival. These studies were further called ‘significant’ ones. Finally, a study was called ‘not significant’ if no statistically significant difference between the two groups was detected.

The association between two continuous variables was measured by the Spearman rank correlation coefficient. Non-parametric tests were used to compare the distribution of the quality scores according to the value of a discrete variable (Mann–Whitney tests for dichotomic variables and Kruskal–Wallis tests for multiple classes variables).

For the quantitative aggregation of the survival results, we measured the impact of COX-2 increase on survival by hazard ratio (HR) between the two survival distributions. For each trial, this HR was estimated by a method depending on the data provided in the publication. The most accurate method consisted of extracting the estimated HR and its standard error (s.e.) from the reported results using two of the following parameters: the HR and its confidence interval (CI) or the *O*−*E* statistic (difference between numbers of observed and expected events), and the log-rank statistic or its *P*-value. If these data were not available, the total number of events, the number of patients at risk in each group and the log-rank statistic or its *P*-value were used to allow for an approximation of the HR estimate. Finally, if the only exploitable data were in the form of graphical representations of the survival distributions, survival rates at some specified times were extracted in order to reconstruct the HR estimate and its variance, with the assumption that the rate of patients censored was constant during the study follow-up (Parmar *et al*, 1998). If this last method was used, three independent persons read the curves to reduce inaccuracy in the extracted survival rates. The individual HR estimates were combined into an overall HR using Peto's method (Yusuf *et al*, 1985), which consisted of using a fixed-effect model assuming homogeneity of the individual true HRs. This assumption was tested by performing *χ*^2^ tests for heterogeneity. If the assumption of homogeneity had to be rejected, we used a random-effect model as a second analysis. By convention, an observed HR<1 implied a better survival for the group with COX-2 increase. This impact of COX-2 on survival was considered statistically significant if the 95% CI for the overall HR did not overlap 1.

When data about global survival of the entire patients' population were available, survival was analysed globally. If authors only reported the results separately for different subgroups, those results corresponding to different cohorts of patients were treated separately in the meta-analysis.

## RESULTS

### Study selection and characteristics

Fourteen publications, published between 1999 and 2005, were eligible for the systematic review (Achiwa *et al*, 1999; Hosomi *et al*, 2000; Khuri *et al*, 2001; Brabender *et al*, 2002; Kim *et al*, 2003; Ab' Saber *et al*, 2004; Araki *et al*, 2004; Lu *et al*, 2004; Yamaguchi *et al*, 2004; Brattstrom *et al*, 2005; Laga *et al*, 2005; Marrogi *et al*, 2005; Richardson *et al*, 2005; Yuan *et al*, 2005). These publications concerned different cohorts of patients. The total number of included patients was 1543, ranging from 53 to 259 patients per study (median: 92). The main characteristics of the 14 eligible publications are reported in Table 1. Nine were dealing with all types of NSCLC, four with ADC and one with large-cell carcinoma. Seven studies only concerned locoregional diseases (two studies concerned stages I–IIIA and four, stages I–IIIB), four only stage I disease and four all stages (I–IV).

Ten studies evaluated COX-2 expression by immunohistochemistry (IHC), two studies assessed COX-2 mRNA overexpression by reverse transcription–polymerase chain reaction (RT–PCR) in real time and the last two studies determined COX-2 gene amplification by *in situ* hybridisation.

Among the 14 studies eligible for the systematic review, four (Hosomi *et al*, 2000; Ab' Saber *et al*, 2004; Brattstrom *et al*, 2005; Marrogi *et al*, 2005) were inevaluable for the meta-analysis owing to a lack of data in the publication, not allowing to calculate the individual HR and its variance.

### Study results report

Six of the 14 studies identified COX-2 overexpression as a poor prognostic factor for survival (with five evaluable for the meta-analysis) whereas one reported that it was a good prognostic factor (evaluable for the meta-analysis). The seven other studies showed no statistically significant impact of COX-2 overexpression on survival (four evaluable for meta-analysis).

Overall, in NSCLC, the rates of COX-2 protein overexpression (detected by IHC), COX-2 mRNA expression (RT–PCR) and COX-2 gene amplification (detected by *in* s*itu* hybridisation) were respectively, 62.4% (number of evaluable tumours *n*=833, 51.7% (*n*=149) and 59.8% (*n*=254). For ADC, IHC and ISH assessments reported were respectively, 69% of COX-2-positive tumours (*n*=368) and 41.2% (*n*=34). The rates of positive tumours by IHC, RT–PCR and *in situ* hybridisation in stage I NSCLC were, respectively, 65% (*n*=240), 50% (*n*=60) and 59.8% (*n*=254).

### Quality assessment

The overall quality score ranged from 36.3 to 66.0% with a median of 51.5%. No statistically significant quality difference was shown between significant and non-significant studies for the global score (median: 55.4 *vs* 48.9%, *P*=0.06). There was also no statistically significant difference between evaluable and non-evaluable studies for meta-analysis in terms of global scores (51.5 *vs* 53.4%, *P*=0.78).

We performed the same analysis of the scores for the 10 studies evaluable for meta-analysis. Their overall quality score ranged between 41.8 and 66%, with a median of 51.5%. As previously observed among eligible publications, there was no statistically significant difference between significant and non-significant studies evaluable for the meta-analysis according to the global score (median of 54.6 *vs* 48.4%, *P*=0.09).

### Meta-analysis

The meta-analysis was performed on 10 studies (1236 patients) dealing with NSCLC, and were shown to have similar methodological scores.

The individual HRs of the 10 evaluable studies were calculated by one of the three methods reported in the Materials and Methods section according to available data. One study reported the data needed to directly calculate the estimated HR (95% CI). In two trials, HR was approximated by the total number of events and the log-rank statistic. For the seven remaining studies, HR had to be extrapolated from the graphical representation of the survival distributions.

**Figure 1:**
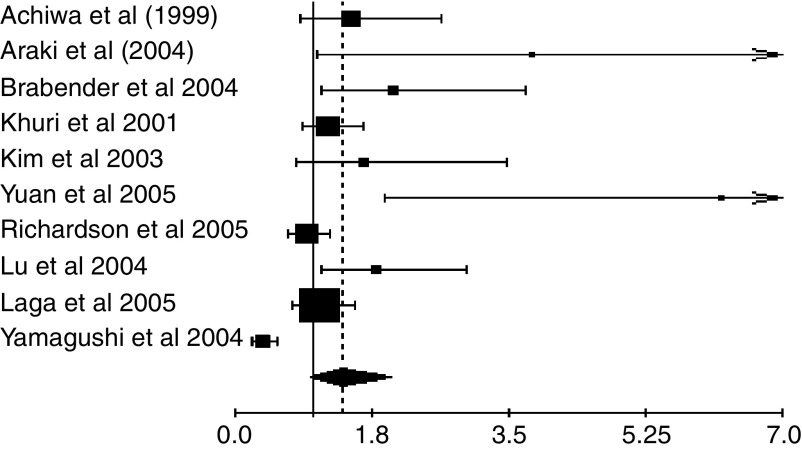
Meta-analysis of the 10 evaluable studies assessing COX-2 in NSCLC. Hazard ratio and 95% CI of survival in studies evaluating COX-2 status in NSCLC. HR>1 implies a survival disadvantage for the group with COX-2 expression. The square size is proportional to the number of patients included in the study. The centre of the lozenge gives the combined HR of the meta-analysis and its extremities give the 95% CI. HR=1.39; CI 95% 0.97–1.99. Total number of patients: 1236.

**Figure 3:**
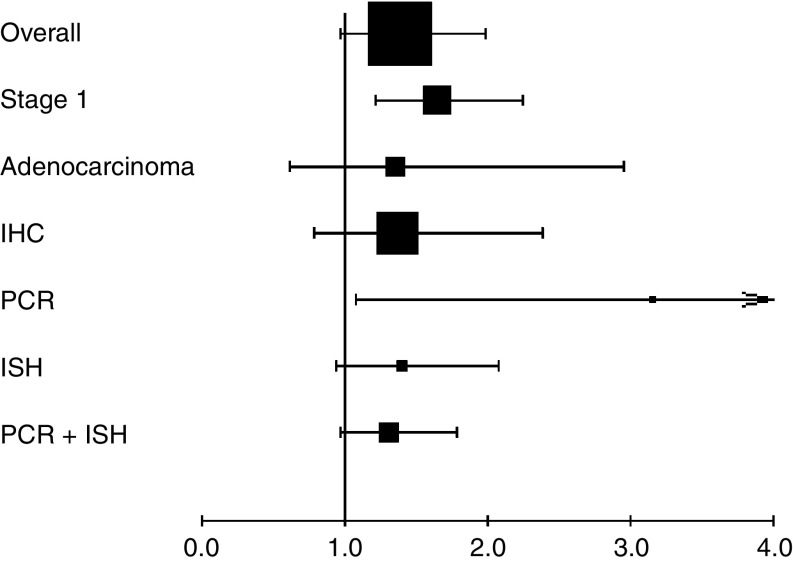
Overall and subgroup analyses. Hazard ratio and 95% CI of survival in studies evaluating COX-2 status in NSCLC. HR>1 implies a survival disadvantage for the group with COX-2 expression. The square size is proportional to the number of patients included in the study and its extremities gives the 95% CI. The Figure 3 shows that there is a trend for a pejorative role of COX-2 as a prognostic survival in NSCLC and that the results become significant (CI not crossing 1) for the subgroups of stage 1 and of RT–PCR.

The results of the meta-analysis are reported in Table 2 and in Figure 3. Overall, COX-2 overexpression was not associated with a significant impact on survival. As the test for heterogeneity was highly significant (*P*<0.001), we also applied a random-effect model in calculating the HR, which was 1.39 (95% CI: 0.97–1.99) (Figure 1).

**Figure 2:**
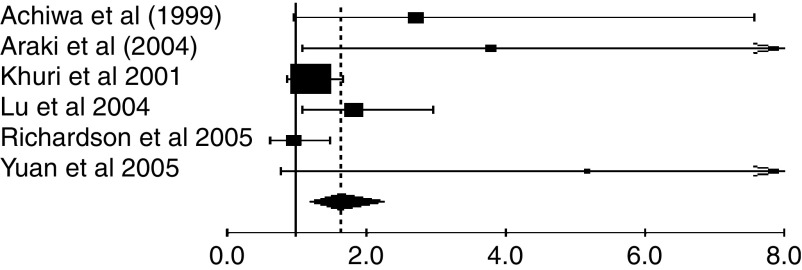
Meta-analysis of the six evaluable studies assessing COX-2 in stage I NSCLC. Hazard ratio and 95% CI of survival in studies evaluating COX-2 status in NSCLC. HR>1 implies a survival disadvantage for the group with COX-2 expression. The square size is proportional to the number of patients included in the study. The centre of the lozenge gives the combined HR of the meta-analysis and its extremities give the 95% CI. Hazard ratio=1.64; CI 95% 1.21–2.24. Total number of patients: 554.

Regarding subgroup analyses (Figure 3), we had the adequate data to aggregate the studies dealing with stage I, with ADC and according to the technique used to detect Cox-2. We first performed an interaction test to assess whether there might be a differential effect of COX-2 according to stage, histology or the technique. We found one significant interaction between COX-2 and stage (*P*<0.01). When we aggregated the six studies (Achiwa *et al*, 1999; Khuri *et al*, 2001; Araki *et al*, 2004; Lu *et al*, 2004; Richardson *et al*, 2005; Yuan *et al*, 2005) giving separate results about stage I NSCLC, the combined HR was statistically significant by using the random-effect model: HR 1.64, 95% CI (1.21–2.24) as there was indeed a significant heterogeneity (*P*=0.04) (Figure 2). We did not observe a statistically significant effect of COX-2 on survival in ADC (five evaluable studies) (Achiwa *et al*, 1999; Araki *et al*, 2004; Yamaguchi *et al*, 2004; Richardson *et al*, 2005; Yuan *et al*, 2005) with HR 1.35 (95% CI 0.62–2.95) (random effect; test of heterogeneity *P*<0.001). We also found one significant interaction between COX-2 and the technique used for its detection (*P*=0.003). The test of heterogeneity was significant for the IHC studies (*P*=0.00001), but neither for RT–PCR studies (*P*=0.1), nor, for ISH studies (*P*=0.18). As the number of studies in the subgroups was small, we only report the HR estimated by the random effect because of a lack of power of the test of heterogeneity. The HRs were the following: for the six studies with IHC (833 patients) 1.06 (95% CI 0.64–1.77), for the two RT–PCR studies (149 patients) 3.15 (1.08–9.21), for the two ISH studies (254 patients) 1.40 (0.94–2.07) and for RT–PCR and ISH studies together (403 patients) 1.31 (0.97–1.76).

## DISCUSSION

The present systematic review of the literature about the impact of COX-2 overexpression on survival in lung cancer found a slight role of COX-2 on overall survival in NSCLC, without not reaching statistical significance. When the analysis was restricted to stage I NSCLC, we observed a statistically significant detrimental effect of COX-2 on survival, suggesting that this prognostic factor could be of importance in early-stage NSCLC. In subgroup analysis according to the different techniques used to detect COX-2, results were only significant with RT–PCR.

The search for a potential prognostic role of COX-2 in survival for patients with lung cancer is based on its frequent overexpression in NSCLC and also on its potential interference with most pathways implicated in lung carcinogenesis. The role of COX-2 in oncogenesis has widely been studied by *in vitro* experiments and by *in vivo* analyses based on animal models. In lung cancer, COX-2 overexpression is associated with microvascular angiogenesis (Masferrer *et al*, 2000) and resistance to apoptosis (Liu *et al*, 1998; Hida *et al*, 2000). Cyclooxygenase-2 overexpression also decreases host immunity (Huang *et al*, 1998) and alters cell adhesion with enhancement of invasion and metastasis (Tsujii *et al*, 1997). Despite all these experimental observations, our meta-analysis failed to demonstrate in univariate analysis a statistically significant impact of COX-2 expression as a prognostic factor for overall survival in patients with NSCLC. In subgroup analysis, we observed a significant effect in stage I NSCLC. Cyclooxygenase-2 overexpression might modify the prognosis of early-stage NSCLC: early lung cancer overexpressing COX-2 would be more aggressive and would have a worse prognosis than those without COX-2 abnormality. These data could be helpful to determine among stage I diseases those who could benefit from a more aggressive treatment. But the present results concerning the prognostic role of COX-2 in stage I NSCLC still need to be confirmed by adequately designed prospective studies with multivariate analysis before a potential clinical application.

It should be noted that COX-2 appears early in oncogenesis for SQCC (Mascaux *et al*, 2005b) as well as for ADC (Hida *et al*, 1998; Wolff *et al*, 1998; Hosomi *et al*, 2000; Hasturk *et al*, 2002). In a previous study (Mascaux *et al*, 2005b), we observed that COX-2 expression increases in bronchial preneoplastic lesions at the stage of severe dysplasia and particularly in clones of cells showing atypia: this suggests an active role of COX-2 in bronchial epithelial cells transformation to malignancy. These data could partially explain the prognostic role of COX-2 at stage I, its impact being lost at later steps because of the potential interaction with many factors.

Our analysis had to deal with heterogeneity problems. There was a highly significant heterogeneity among the 10 evaluable studies included in the meta-analysis. This could be explained by the type of patients and the disease characteristics, or by the diversity in the techniques used to identify alteration of COX-2 status. Only six evaluable studies used IHC, two ISH and two RT–PCR. The results of subgroup analysis according to the technique used to detect COX-2 support this hypothesis. Results for the six IHC studies were not significant and a high heterogeneity was detected between the studies (*P*=0.003). This heterogeneity could be explained by the fact that the technique of IHC is not comparable among the six studies. The primary antibodies were different and so was the revelation protocols, and different levels of positivity (0, 10, 50%, different scores combining intensity and percentage, intensity only) were used. As another example, when ISH and RT–PCR (two different techniques assessing RNA) studies were aggregated together, the heterogeneity increased (*P*=0.03) as compared with ISH alone (*P*=0.18) or RT–PCR alone (*P*=0.1), and with only a few studies, the results were statistically significant for the RT–PCR subgroup, which is the most standardised technique. It is thus very important to use a well-defined and well-standardised technique to be reproducible for the evaluation of biological markers. Particularly, the protocol of IHC should be the same between different laboratories (same antibody, same revelation protocol (pH and compounds of the solutions, heating method etc) and same criteria of evaluation for the positivity of the marker) so that the results could be compared and eventually, aggregated.

Some other biases could be due to the methodology used to perform our systematic review. We performed a methodological assessment of the studies to avoid some selection biases (more detailed reports of significant trials), as we performed in prior studies about biological prognostic factors in lung cancer (Steels *et al*, 2001). The absence of a detectable difference in quality score between significant and non-significant studies, and between evaluable and non-evaluable studies, encourages us to perform a quantitative aggregation (meta-analysis) of the results of the individual trials. However, in the present review, numbers of studies are small, preventing us to analyse any potential difference between significant and non-significant, or evaluable and non-evaluable studies. However, this approach does not prevent all potential biases. Publication bias, choice of language, selection of fully published studies only, method of extrapolation of HR, validity of a meta-analysis based on systematic review of the literature as compared with those based on individual data were already discussed in our previous papers (Steels *et al*, 2001).

Some eligible trials had to be excluded from the meta-analysis because they did not provide sufficient data on survival. Among the four excluded studies, only one (25%) was statistically significant, whereas a higher proportion of the studies evaluable for the meta-analysis were significant (60%). It is known that negative studies are less frequently published or, if they are, with less detailed results, making them less assessable. The methodological quality of trials, according to the global score, was not significantly different between evaluable and non-evaluable studies for the quantitative aggregation of individual survival results. Nevertheless, such an approach does not fully protect a potential bias owing to the impossibility taking into account all the studies with negative or non-significant results.

Our meta-analysis is based on published data collected by a systematic review of the literature and can only be performed by univariate analysis. This is a limit to this type of work, which appears thus as a preliminary step before performing multivariate studies. Many interesting data arise from multivariate analyses and particularly from proteomic and genomic wide screen analysis, which is probably the way of the future. But if microarrays is an interesting technique providing very meaningful data, it should be kept in mind that it remains a research screening technique and that it could not be applied in routine because of the high price.

It should also be noted that COX-2 expression increases in patient treated by taxanes (Subbaramaiah *et al*, 2003; Altorki *et al*, 2005), providing an argument to treat patients with lung cancer by an association of taxanes and anti-COX-2 drugs. The studies analysing COX-2 expression after a specific treatment were not included in this meta-analysis because treated and untreated tumours do not have the same biological behaviour and should not be aggregated together. This topic, COX-2 expression in pretreated lung tumours, should be the topic of another systematic review.

In conclusion, when all stages and histologies are considered, there is a trend for COX-2 overexpression as a prognostic factor for survival in patients with NSCLC, but there is a high heterogeneity between the studies and these results are not statistically significant. Interestingly, our meta-analysis showed with more evidence that COX-2 has a detrimental effect on survival in stage I NSCLC. This prognostic role of COX-2 at earliest stage of NSCLC could be of clinical interest in the selection of the patients eligible for induction or adjuvant chemotherapy. Hazard ratio was also significant for the studies using RT–PCR and not for those using IHC, suggesting that a better standardisation of the technique to define and to detect COX-2 positivity is required to the generalisability of the results. Our results need to be confirmed by an adequately designed prospective study and the exact role of COX-2 overexpression needs to be determined by an appropriate multivariate analysis taking into account the classical well-defined (at the moment of the study) prognostic factors for lung cancer such as PS, stage, age, sex, weight loss.

## Figures and Tables

**Table 1:** Main characteristics and results of the eligible studies

**First author**	**Year**	**Histology**	**Stage**	**N pts**	**Laboratory method**	**Antibody for IHC, dilution**	**Definition of Cox-2 positivity**	**HR estimation**	**Evaluable**	**Survival results**
Ab' Saber	2004	LC	I–IIIB	61	IHC	Dako, 50	Score 1	No data	No	Negative
Achiwa	1999	ADC	I–IIIB	130	IHC	IBL, 25	I>ref	Surv. curves	Yes	NS
Araki	2004	ADC	I	71	IHC	Cayman, 500	>10%	Log rank	Yes	Negative
Brabender	2002	NSCLC	I–IIIA	89	RT-PCR		Ratio ref.	Surv. curves	Yes	Negative
Brattstrom	2004	NSCLC	I–IV	53	IHC	SantaCruz, 1000	>67% + I	No data	No	NS
Hosomi	2000	ADC	I–IIIB	87	IHC	IBL, 50	>10%	No data	No	NS
Khuri	2001	NSCLC	I	160	ISH		1%	Surv. curves	Yes	NS
Kim	2003	NSCLC	I–IIIA	84	IHC	Cayman, 100	Score 2	Surv. curves	Yes	Negative
Laga	2005	NSCLC	I–IV	259	IHC	Cayman, 150	Score 3	Surv. curves	Yes	NS
Lu	2004	NSCLC	I	94	ISH		1%	HR	Yes	Negative
Marrogi	2000	NSCLC	I–IV	106	IHC	SantaCruz, 100	Score 4	No data	No	NS
Richardson	2005	NSCLC	I–IIIA	172	IHC	SantaCruz, 400	>50%*	Surv. curves	Yes	NS
Yamaguchi	2004	ADC	I–IIIB	117	IHC	Transduc.,100	Score 5	Log rank	Yes	Positive
Yuan	2005	NSCLC	I–IV	60	RT-PCR		Ratio ref.	Surv. curves	Yes	Negative

Abbreviations:ADC, adenocarcinoma; HR, hazard ratio; IBL, Immuno-biological laboratory; IHC, immunohistochemistry; ISH, *in situ* hybridisation; N pts, number of patients; NSCLC, non-small–cell lung cancer; ref, reference; RT–PCR, reverse transcriptase–polymerase chain reaction; surv. curves, survival curves; LC, large cell; NS: non significative; Transduc: Transduction; score 1: score from 0 to 8 without any explanation, positive ⩾2, I: intensity; score 2, 3, 4, 5: different scores with combination of percentage of positives cells and intensity,

* >50%: thresehold=median of positivity for COX-2, which was 50%. HR estimation: description of the methods used to estimate the individual HR according to the three different methods described in the statistics methodology (see statistical methods).

**Table 2:** Meta-analysis: HR value in NSCLC subgroups according to histology, stage

	**Nb**	**Patients**	***χ*^2^ heterogeneity test**	**Random effects HR (95% CI)**
Overall	10	1236	***P*=0.000001**	1.39 (0.97–1.99)
Stage I disease	6	554	***P*=0.04**	**1.64 (1.21–2.24)**
Adenocarcinoma	5	402	***P*=0.000001**	1.35 (0.62–2.95)
IHC	6	833	***P*=0.00001**	1.06 (0.64–1.77)
RT–PCR	2	149	*P*=0.1	**3.15 (1.08–9.21)**
ISH	2	254	*P*=0.18	1.40 (0.94–2.07)
RT–PCR + ISH	4	403	***P*=0.03**	1.31 (0.97–1.76)

Abbreviations: HR, hazard ratio; IHC, immunohistochemistry; ISH, *in situ* hybridisation; Nb, number of studies; RT–PCR, reverse transcriptase–polymerase chain reaction.

Statistically significant results are in bold.
